# Influence of academic year on dental student’s knowledge about syphilis and its oral manifestations

**DOI:** 10.1590/1807-3107bor-2026.vol40.043

**Published:** 2026-07-31

**Authors:** Antonio Carlos PACHECO FILHO, Karina Tonini dos Santos PACHECO, Ana Carla Layber PORTO, Clea Adas Saliba GARBIN

**Affiliations:** (a) Universidade Estadual Paulista – Unesp, School of Dentistry, Postgraduate Program in Preventive and Social Dentistry, Araçatuba, SP, Brazil.; (b) Universidade Federal do Espírito Santo - UFES, Dentistry, Postgraduate Program in Dental Sciences, Vitória, ES, Brazil.

**Keywords:** Syphilis, Students, Dental, Oral Manifestations

## Abstract

This study aimed to investigate the influence of the year in the course on dental students’ knowledge about syphilis and its oral manifestations. A cross-sectional study was conducted with dental students at the Federal University of Espírito Santo, Brazil, who were divided into two groups: earlier years of the Dentistry program (second and third years) and later years (fourth and fifth years). Data were collected using a questionnaire with 16 questions addressing knowledge of the main characteristics of syphilis. The Chi-square test (or Fisher’s exact test with Yates correction) was used to assess the association between variables, with a significance level of 5%. The prevalence ratio (PR) and the 95% confidence interval (95% CI) were used as measures of effect size. Overall, 171 students were invited, and 169 participated in the study. Only 40 (23.7%) students answered correctly about the stages of the disease. Nearly all participants answered that syphilis presents oral manifestations; however, only 44 (26%) answered the question correctly. Regarding differential diagnoses, only 63 (37.3%) reported knowledge of the subject. The direction of the bivariate analysis was inverse to the year of study, indicating a possible association between students enrolled in the early years of the Dentistry program and better knowledge about etiologic agents (p < 0.001), clinical manifestations (p < 0.001), disease stages (p < 0.001), oral manifestations (p < 0.001), differential diagnosis (p = 0.019), and drugs (p = 0.001) related to the disease compared with students enrolled in the later years.

## Introduction

Syphilis is a sexually transmitted infection (STI) caused by *Treponema pallidum*. It is a public health concern and is among the most common communicable diseases, affecting the health and lives of people worldwide. In 2019, the global incidence was 14.1 million cases, and the global prevalence was 49.7 million cases, according to the Global Burden of Disease Study 2019 (GBD 2019).^1,2^ Data from the Centers for Disease Control and Prevention (CDC) show that, in the United States, men who have sex with men remain more affected by syphilis, accounting for 54% of primary and secondary syphilis cases, which may be associated with HIV co-infection.^3^

The situation of syphilis in Brazil is similar: the number of cases is concerning. The detection rate of acquired syphilis, a condition of compulsory notification since 2010, increased from 9.3 cases per 100,000 inhabitants in 2011 to 99.2 cases per 100,000 inhabitants in 2022.^4^

The disease has four distinct stages, each characterized by specific symptoms, clinical manifestations, and infectivity levels: primary, secondary, tertiary, and latent. Clinical manifestations may appear in the oral cavity and perioral region, and primary and secondary lesions are highly contagious.^5^ For this reason, dentists often make the diagnosis and play a crucial role in effective, control, and treatment by identifying signs and symptoms, guiding patients on procedures, providing treatment support, and ensuring follow-up.^5^

Given the importance of academic training that enables dentists to join multiprofessional teams for the diagnosis and treatment of STIs^6^, and that knowledge may influence attitudes and behaviors,^7^ the aim of this study was to investigate the influence of the year in the course on dental students’ knowledge about syphilis and its oral manifestations.

## Methods

### Study design

This cross-sectional study was conducted with dental students from the Federal University of Espírito Santo (UFES), Brazil, following the Strengthening the Reporting of Observational Studies in Epidemiology (STROBE) guidelines^8^ for cross-sectional studies, to assess knowledge about syphilis and its oral manifestations among these students. UFES is located in the capital of Espírito Santo, in southeastern Brazil, and is the only public university in the state. The research was conducted ethically in accordance with the Declaration of Helsinki (World Medical Association). This project was approved by the local Ethics Committee (89560418.6.0000.5060).

### Participants

Research subjects were included in the study based on two criteria: agreeing to participate in the study and being present in the classroom on the day the study was carried out. All participants who agreed to participate signed an informed consent form.

The sample included all students enrolled in the second semester of the second (27), third (65), fourth (55), and fifth (44) years, totaling 171 students. These students were divided into two groups for association analyses: earlier years of the Dentistry program (second and third years) and later years (fourth and fifth years).

Students in the first year and the first semester of the second year (third term) were excluded because they had not taken the Stomatology course, which officially covers the content discussed in this study and is offered in the fourth semester of the Dentistry program. In addition, students who were absent on the day the questionnaire was administered, who did not agree to participate, or who did not sign the consent form were excluded.

### Data collection and variables

Data were collected through a structured questionnaire consisting of closed-ended questions, specifically designed for the research. Students did not know they would be asked about syphilis and its oral manifestations on the day they were approached. The questionnaire was administered collectively in the classroom at a time considered appropriate and convenient for the students, with privacy and confidentiality of the information collected guaranteed.

The scientific literature^9-12^ and guides from the Brazilian Ministry of Health^13^ and the Sexually Transmitted Infections Treatment Guidelines^14^ were consulted to build the questionnaire. The final version contained 16 questions, which were sufficient to cover all important aspects of knowledge about syphilis: identification (sex, age), school year, and aspects related to knowledge about syphilis, including its definition, etiological agent, modes of transmission, clinical manifestations, disease stages, oral manifestations, differential diagnoses, and drugs used for treatment. Two specialists in Stomatology, who are also professors of dentistry, were then consulted to provide their opinions on the instrument.

The response options for the multiple-choice questions were defined using two main criteria. First, the alternatives were developed based on specialized scientific literature, which provided the technical content needed to formulate correct and conceptually valid options. Second, the alternatives deliberately included incorrect but plausible answers, based on misconceptions frequently observed in clinical practice and reported in previous studies.^16,17^ This methodological approach aimed to assess both the students’ correct theoretical knowledge and their ability to recognize and avoid common conceptual errors.

The independent variable of the study was the year of enrollment in the Dentistry program, grouped into early years (2nd and 3rd years) and final years (4th and 5th years). The dependent variables concerned students’ knowledge about syphilis and its oral manifestations, including knowledge of the etiological agent of syphilis, modes of transmission, clinical manifestations, stages of the disease, oral manifestations, differential diagnoses, and drugs used in treatment. Each variable was analyzed dichotomously (yes/no or correct/incorrect) based on the participants’ responses.

After the theoretical construction and expert review, the questionnaire was piloted with first-year dental students who were not part of the final study sample, to generate preliminary empirical evidence of validity. This stage allowed assessment of item performance, clarity of questions and response options, and internal consistency of the instrument. The pilot results indicated that respondents adequately understood the items, encountered no significant difficulties during completion, and that the questions behaved as expected, with no need for item exclusion or revision. These findings provide initial empirical support for the instrument’s validity and reinforce its suitability for assessing knowledge about syphilis among dental students.

### Statistical analysis

Questionnaire data were entered into IBM SPSS Statistics for Windows 19.0 by two researchers, maintaining confidentiality. An independent researcher reviewed 10% of the database entries to validate them for analysis. Data were statistically analyzed using absolute and relative frequencies. A bivariate analysis was then performed to assess associations among variables, using the Chi-square test or Fisher’s exact test with Yates correction, at a 5% significance level, relating knowledge about syphilis to the educational level of dental students. The prevalence ratio (PR) and 95% confidence interval (95% CI) were used as measures of effect size.

## Results

In the univariate analysis, the sociodemographic independent variables characterizing the sample and the dependent variables related to knowledge were described. Of the 171 students, 169 completed the questionnaires (those present in the classroom on the day of administration who agreed to participate), resulting in a response rate of 98.8% (Table 1). Only two students did not participate because they were absent that day.

**Table 1:** Characteristics of study participants

Variables	n (%)
Sex	
Female	129 (76.3)
Male	40 (23.7)
Age (years)	
< 25	139 (82.2)
25–29	26 (15.4)
30–34	2 (1.2)
35–39	2 (1.2)
Educational level	
Earlier years of dentistry program (second and third years)	86 (50.9)
Later years of dentistry program (fourth and fifth years)	83 (49.1)

All students reported knowing about syphilis, and 167 (98.8%) received information about the disease during their undergraduate course. Of all students, 65 (38.5%) reported that one of the most common syphilis lesions is a hard chancre. Only 40 (23.7%) students correctly answered the question about the stages of the disease. Almost all participants stated that syphilis has oral manifestations; however, only 44 (26%) correctly answered the question, indicating that the appearance of a chancre is common in the primary stage of the disease (Table 2).

**Table 2:** Knowledge about syphilis and oral manifestations among dental students.

Questions about syphilis	Students n (%)
Forms of transmission	166 (98.2%)
Sexual contact	160 (94.7%)
Vertical	124 (73.4%)
Dental office	101 (59.8%)
Etiological agent	
*N. gonorrhoeae*	5 (2.9%)
*C. trachomati*	6 (3.5%)
*T. pallidum*	135 (79.9%)
*T. vaginalis*	16 (9.5%)
*H. ducreyi*	0 (0%)
Clinical manifestations	84 (49.7%)
General clinical manifestations	
One of the most common lesions is soft chancre	10 (5.9%)
It is not possible to observe skin changes	2 (1.2%)
One of the most common lesions is hard chancre	65 (38.5%)
Thrush is commonly observed	5 (3.0%)
Disease stages	
The disease is more contagious in the third stage with the presence of hard chancre	29 (17.1%)
The third stage may not show signs	40 (23.7%)
It is not possible to observe skin changes such as spots	5 (2.9%)
There is no risk of contamination in the primary phase	6 (3.5%)
Oral clinical manifestations	167 (98.8%)
There is no risk of contamination in the primary stage	23 (13.6%)
In the secondary stage, the appearance of syphilitic gumma is common	34 (20.1%)
In the tertiary stage, the appearance of hard chancre is common, which is the healing of lesions of primary and secondary stages	62 (36.7%)
The appearance of hard chancre is common in the primary stage	44 (26.0%)
Knowledge on differential diagnoses	63 (37.3%)
Lichen Plan	16 (9.5%)
Polymorphic erythema	10 (5.9%)
Not a differential diagnoses of syphilis	
Soft chancre	12 (7.1%)
Hairy Leukoplakia	12 (7.1%)
Herpes	14 (8.3%)
Main medication	
Penicillin	109 (64.5%)
Azithromycin	12 (7.1%)
Erythromycin	3 (1.8%)
Fluconazole	15 (8.9%)
Acyclovir	23 (13.6%)

Response options (correct answer is in bold).

Regarding the different diagnoses, only 12 (7.1%) correctly responded the question (Hairy Leukoplakia). Moreover, 109 (64.5%) knew that the main form of treatment is penicillin (Table 2).

In the bivariate analysis, there was a statistically significant association between the students’ educational level in the Dentistry program and their knowledge across all domains. Students in the early years of the program demonstrated better knowledge about the disease compared to those in the final years (Table 3). This analysis aimed to identify possible associations between the variables in order to determine, in cases of insufficient student knowledge on the subject, at which stage of the undergraduate program potential gaps in the learning process might be occurring.

**Table 3:** Associations between correct responses regarding knowledge about syphilis and educational level of dental students.

Variabes	Earlier years of		Later years of		PR	95%CI	p-value
	dentistry program		dentistry program				
Knowledge about syphilis	Incorrect n (%)	Correct n (%)	Incorrect n (%)	Correct n (%)			
Etiological agent	6 (7.1)	79 (92.9)	27 (32.5)	56 (67.5)	0.73	0.62–0.85	< 0.001
Clinical manifestations	34 (40.0)	51 (60.0)	69 (83.1)	14 (16.9)	0.42	0.31–0.57	< 0.001
Disease stages	47 (58.0)	34 (42.0)	77 (92.8)	6 (7.2)	0.45	0.34–0.58	< 0.001
Oral manifestations	47 (55.3)	38 (44.7)	77 (92.8)	6 (7.2)	0.44	0.34–0.57	<0.001
Differential diagnosis	75 (88.2)	10 (11.8)	81 (97.6)	2 (2.4)	0.58	0.43–0.78	0.019
Drugs	20 (23.5)	65 (76.5)	39 (47.0)	44 (53.0)	0.57	0.39–0.84	0.001

Regarding the etiological agent, a higher proportion of correct answers was observed among students in the earlier years (92.9%) compared to those in the later years (67.5%) (PR = 0.73; 95%CI: 0.62–0.85; p < 0.001). For clinical manifestations, 60.0% of students in the earlier years and 16.9% in the later years answered correctly (PR = 0.42; 95%CI: 0.31–0.57; p < 0.001). A similar pattern was observed for knowledge of disease stages, with correct responses from 42.0% of students in the earlier years and 7.2% in the later years (PR = 0.45; 95%CI: 0.34–0.58; p < 0.001).

In the domain of oral manifestations, 44.7% of students in the earlier years and 7.2% in the later years answered correctly (PR = 0.44; 95%CI: 0.34–0.57; p < 0.001). For differential diagnosis, the proportion of correct responses was low in both groups, with 11.8% in the earlier years and 2.4% in the later years (PR = 0.58; 95%CI: 0.43–0.78; p = 0.019). Finally, regarding drugs used for the treatment of syphilis, 76.5% of students in the earlier years and 53.0% in the later years answered correctly (PR = 0.57; 95%CI: 0.39–0.84; p = 0.001).

## Discussion

Oral syphilis is considered a public health issue in Brazil, and dentists’ knowledge is important for improving diagnosis and treatment. Results from this study showed that, in general, dentistry students have limited knowledge about syphilis and its oral manifestations. Although all students who claimed to know about syphilis had studied the subject during their undergraduate program, less than half correctly identified the clinical manifestations of the disease. In 2023, Medeiros et al.^6^ reported that senior dental students’ performance was unsatisfactory in a study evaluating knowledge of the prevention, diagnosis, and treatment of oral manifestations of syphilis.

From this perspective, few students were aware of the oral manifestations of syphilis. Consistent with this study, Wu et al. found that when investigating knowledge about occupational blood-borne pathogens among Chinese dentistry students, less than half of the participants could recognize the main oral manifestations of syphilis.^7^

Recognizing these oral manifestations is a crucial responsibility for dentists, as oral lesions are highly contagious. Accurate diagnosis supports proper management, reduces the chain of infection, and reduces the risk of transmission to healthcare professionals.^15^ It is worth noting that infection through clinical dental practice due to exposure to contaminated blood or saliva was the least reported form of transmission by students. This typically occurs when proper professional practices are not followed.^5,16^

In addition, a small percentage of students reported knowledge of the differential diagnoses of oral manifestations of the disease. Studies have shown that diagnosing oral manifestations of syphilis is challenging for professionals because these manifestations have a variety of clinical appearances.^11,17,18^ The similarity of some characteristics to other conditions is concerning, and if diagnosis is not made during the primary and secondary stages, the patient is at risk of complications related to the tertiary stage.^17^

Our study showed that the direction of the bivariate analysis was inverse to the year of study, indicating a possible association between students enrolled in the early years of the Dentistry program and better knowledge about etiologic agents, clinical manifestations, disease stages, oral manifestations, differential diagnosis, and drugs compared with students enrolled in the last two years.

In the basic cycle, students take the Stomatology course, which is responsible for addressing the oral manifestations of sexually transmitted infections. This may help explain the possible association between students in the early years of the Dentistry program and the better knowledge about syphilis found in the results, as the topic may have been studied recently. Furthermore, students may have limited or no contact with patients presenting with oral manifestations of syphilis as they progress to the later years of the program, which may lead to a lack of recognition of the disease’s clinical signs.

With the development of the new 2021 National Curriculum Guideline for undergraduate Dentistry programs in Brazil, our institution created a new pedagogical project, approved in 2024 (Cepe/Ufes 2024), primarily aimed at integrating basic knowledge with clinical application (DCN). Furthermore, the Stomatology course now consists of 75 hours, with 60 hours dedicated to clinical practice in dental care and extension activities, compared to the previous 30 hours of practice, which already accounted for 50% of the total course load.

It is worth noting that Ufes is the only public institution offering Dentistry in the state of Espírito Santo, admitting 60 students per year and accounting for approximately 25% of the professionals registered with the state’s Regional Dental Council (CRO). Consequently, it represents a significant portion of the professionals practicing in the state after graduation. Given the importance of this institution in providing access to dental education in Espírito Santo, this study reinforces the relevance of the curricular improvements undertaken, which make dental care more effective in the diagnosis, follow-up, and treatment of syphilis in patients seeking care in the state.

Unlike this research, studies that investigated the knowledge of dental students about HIV/AIDS^22-24^ and occupational exposure to blood-borne pathogens^25,26^ showed that knowledge of some aspects was greater among students with a higher educational level. Keser et al. justified this finding by noting that the older group may have gained more experience over the years and had exposure to a larger number of patients compared to the younger group.^22^ Brailo et al.^26^ reported that this finding can be explained by the fact that, at the beginning of clinical practice phase in each department, students are reintroduced to this content again.

Although students take the Oral Medicine course in the first years of the Dentistry program, it is essential that this content is revisited in other courses throughout the undergraduate program, so that students remain aware of all aspects of the disease, regardless of their current year of training.

*Treponema pallidum* can be transmitted through exposure to infected blood and body fluids, such as saliva.^11,27^ This possibility of non-sexual transmission places dentists among the healthcare professionals most at risk of contamination, as accidental contact with saliva and blood may occur during clinical practice. The risk is even greater considering that 9 out of 10 patients with syphilis do not present any clinical manifestations, although they remain infectious or unaware of the infection.^3,16^

One notable fact was that studies on knowledge of blood-borne pathogen transmission among dental students by Myers et al.^25^ and Brailo et al.^26^ did not address syphilis.^25,26^ The study by Wu et al.^7^ included syphilis, likely due to its increasing prevalence worldwide. Syphilis has reemerged in recent years, so many clinicians do not consider it in their differential diagnosis of oral lesions.^28^

Fewer participants than expected recognized penicillin as the drug of choice for treatment. Penicillin became widely accepted as an effective treatment for syphilis,^11^ and the success in controlling the disease may have created a significant gap in medical and dental education.

The main study limitations because was that it was conducted at only one institution. Our findings, along with the increasing number of new cases of the disease worldwide, highlight the need to train dental professionals in early diagnosis, effective treatment, and follow-up of syphilis cases. Since the main mode of transmission is sexual, diagnosis and treatment must consider socio-cultural and ethical factors. The disease affects the lives of those impacted, and this issue should also be addressed in undergraduate courses.^29^

It is important to foster students’ interest not only in the clinical aspects of the disease but also in care-related issues. Enhancing students’ knowledge can effectively increase their willingness to treat patients.^7^ However, it is necessary to seek educational methods that can improve students’ experience and learning skills.

## Conclusion

Participants showed important gaps in their knowledge about syphilis, particularly regarding differential diagnosis and general and oral clinical manifestations. A possible association was observed between knowledge and students’ educational level during the undergraduate course, with higher knowledge levels among students in the earlier years of the program. Our results support the recommendation to improve or include the approach to syphilis and its oral manifestations in the curricula of Dentistry Education Institutions. We believe this is an important intervention that can help control and prevent the disease in areas where it is prevalent.

## Data Availability

The datasets generated during and/or analyzed during the current study are available from the corresponding author on reasonable request.
